# Structure-guided optimization of small molecule c-Abl activators

**DOI:** 10.1007/s10822-014-9731-5

**Published:** 2014-02-27

**Authors:** Xuan Hong, Ping Cao, Yoshiaki Washio, Graham Simpson, Nino Campobasso, Jingsong Yang, Jennifer Borthwick, George Burton, Julien Chabanet, Sophie Bertrand, Helen Evans, Robert J. Young, Junya Qu, Hu Li, Josh Cottom, Paris Ward, Hong Zhang, Thau Ho, Donghui Qin, Siegfried Christensen, Martha S. Head

**Affiliations:** 1 Platform Technology and Science, GlaxoSmithKline, Collegeville, PA USA; 2 Platform Technology and Science, GlaxoSmithKline, Stevenage, UK; 3 Oncology Research and Development, GlaxoSmithKline, Collegeville, PA USA; 4 Virtual Proof of Concept Discovery Performance Unit, GlaxoSmithKline, King of Prussia, PA USA

**Keywords:** c-Abl, Myristoyl, Kinase activation, Kinase activators, Multi-fragment search, Docking

## Abstract

c-Abl kinase is maintained in its normal inactive state in the cell through an assembled, compact conformation. We describe two chemical series that bind to the myristoyl site of the c-Abl kinase domain and stimulate c-Abl activation. We hypothesize that these molecules activate c-Abl either by blocking the C-terminal helix from adopting a bent conformation that is critical for the formation of the autoinhibited conformation or by simply providing no stabilizing interactions to the bent conformation of this helix. Structure-based molecular modeling guided the optimization of binding and activation of c-Abl of these two chemical series and led to the discovery of c-Abl activators with nanomolar potency. The small molecule c-Abl activators reported herein could be used as molecular tools to investigate the biological functions of c-Abl and therapeutic implications of its activation.

**Electronic supplementary material:**

The online version of this article (doi:10.1007/s10822-014-9731-5) contains supplementary material, which is available to authorized users.

## Introduction

Protein kinases have been an intensively pursued drug target family due to their involvement in many disease indications such as cancer, inflammation, autoimmune diseases and metabolic disorders [1]. Several small molecule kinase inhibitors have been approved by the FDA for clinical use [2–8]. These drugs and the majority of kinase inhibitors target the ATP binding site on the catalytic kinase domain, thereby blocking binding of ATP and phosphorylation of substrates, and ultimately attenuating downstream physiological effects of kinases in disease states.

In addition to intense research on inhibiting kinase activity, there have also been efforts to identify small molecules that up-regulate kinase activity [9]. Successful kinase activators would not only enrich our understanding of the regulation mechanism of protein kinase activity but would potentially be of therapeutic interest. We present herein a successful structure-guided optimization of small molecule c-Abl activators that could serve as molecular tools to investigate therapeutic implications of c-Abl activation and also provide insight into the mechanisms behind c-Abl activation at a molecular level.

c-Abl, the cellular form of the Abelson leukemia virus tyrosine kinase, is involved in a broad range of cellular processes, although many aspects of its functions remain poorly understood. Among what is known, c-Abl is believed to play a role in cell cycle regulation, stress responses to DNA damage, oxidation and ionization, and signal transduction induced by extracellular stimuli [10–13]. Evidence suggests that c-Abl activation may lead to the inhibition of mammary tumorigenesis and breast cancer cell mobility and invasiveness [14, 15]; c-Abl activation may also have a role in the process of myelopoiesis [16, 17].

c-Abl is a non-receptor tyrosine kinase and contains three structural domains in its N-terminal portion: SH3, SH2, and catalytic kinase domains. There are two human c-Abl isoforms, c-Abl 1a and 1b, derived from alternative splicing events [18]. Although both isoforms have a N-terminal cap region, the cap region of c-Abl 1a is 19 amino acids shorter than that of c-Abl 1b. In addition, c-Abl 1b is amidated to a 14-carbon saturated fatty acid, myristic acid at the end of its N-terminal cap, whereas c-Abl 1a is not. c-Abl is normally maintained in its inactive state in the cell; however, in patients with Chronic Myelogenous Leukemia (CML), the gene encoding c-Abl is fused with the breakpoint cluster region (BCR) gene, resulting in the formation of the fusion protein BCR–Abl. BCR–Abl is constitutively active, leading to abnormal proliferation of white blood cells. When fused with BCR, all of the c-Abl domains—SH3, SH2, and kinase domains—are retained except for the N-terminal cap, which suggests that the cap region of the wild-type protein may negatively regulate c-Abl.

**Fig. 1:**
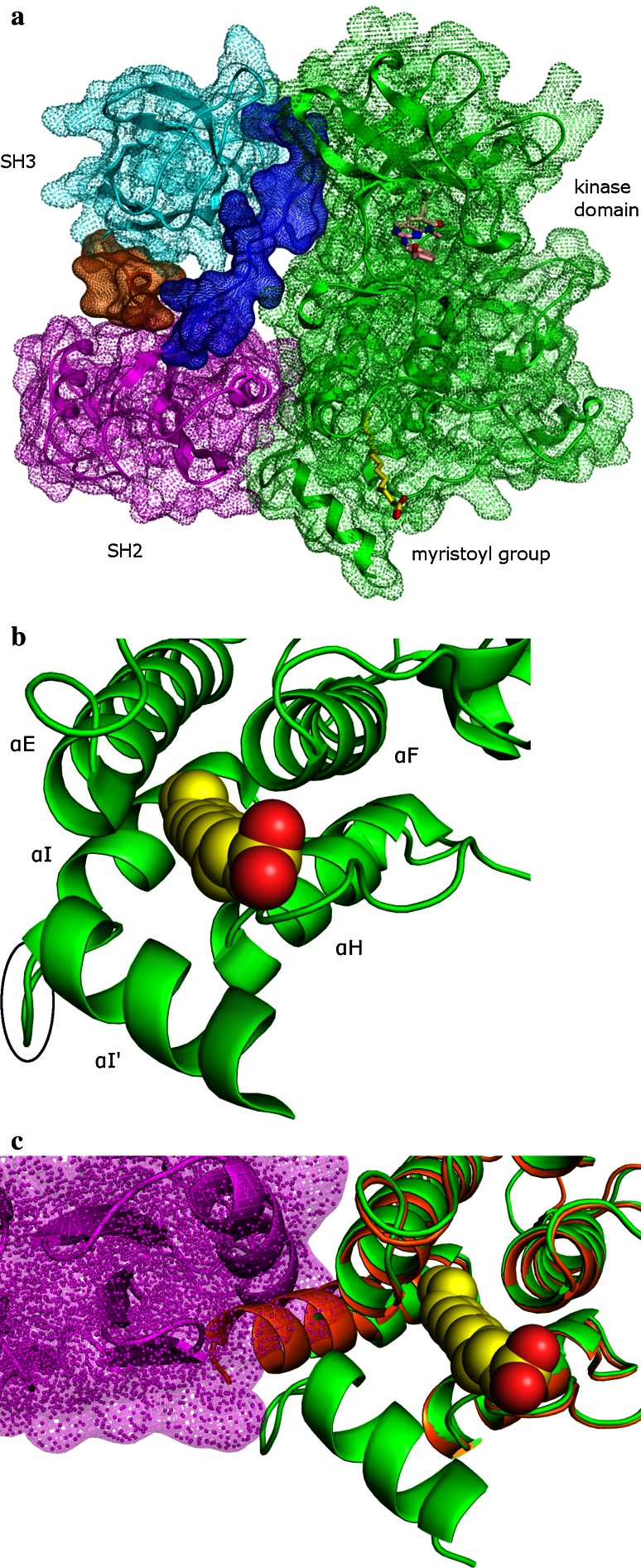
**a** Full view of the assembled c-Abl structure (PDB code: 1OPK). The SH3, SH2, and the kinase domains are shown in *cyan*, *magenta*, and *green*, respectively. The linker between the SH3 and the SH2 domains is in *brown*, whereas the linker between the SH2 and the kinase domains is in *blue*. The small molecule bound to the ATP-binding site is PD166328 [19] and is shown in *pink* carbon. The myristoyl group is in *yellow* carbon. **b** The myristoyl group in the c-Abl myristoyl site; the myristoyl group is shown in space-filling format and in *yellow* carbon. The bend between αI and αI′ (Met515–Ser519) is highlighted by a *black circle*. **c** Overlay of the assembled c-Abl structure with the myristoyl-free c-Abl kinase domain (PDB code: 1M52). The myristoyl-free c-Abl is shown in brown. The van der Waals surface of the SH2 domain is shown in *magenta* to illustrate the steric clash between the SH2 domain and the straight from of the C-terminal helix in the myristoyl-free c-Abl. All of the figures presented in this paper were generated using PyMol

Crystal structures of the N-terminal portion of c-Abl 1b have been determined [19, 20]. Due to the availability of these structures and the mechanism of c-Abl activation by the small molecules that will be described in this paper, we will focus on the c-Abl 1b isoform of the protein and refer to c-Abl 1b simply as c-Abl. Crystal structures of the c-Abl N-terminal portion show that autoinhibited c-Abl adopts a compact conformation in which the SH2 domain docks onto the kinase domain and the SH3 domain interacts with both the kinase domain and the linker between the SH2 and the kinase domains (Fig. 1a). Although the electron density is not defined for part of the N-terminal cap, the electron density of the myristoyl group is clear and shows that this group inserts itself like a latch into a deep and largely hydrophobic pocket located in the C lobe of the kinase domain. This pocket, often called the myristoyl site, is largely composed of helices αE, αF, αH, αI and αI′ (Fig. 1b). In the autoinhibited structures, a bent conformation of five amino acids (Met515–Ser519) introduces a kink between αI and αI′. Alternate conformations have been seen in the αI/αI′ region when the myristoyl site is empty, with αI and αI′ forming a single straight helix (Fig. 1c) [21]. (For simplicity, αI and αI′ will be referred to as the C-terminal helix in the rest of this paper.) Under the bent conformation, the SH2 domain docks against the kinase domain and has extensive interactions with the C-lobe of the kinase domain, including a hydrogen bonding network with the C-terminal helix [19], while a straight C-terminal helix geometry would clash with the SH2 domain (Fig. 1c). As a result, the SH2 domain would no longer be able to retain close contact with the kinase domain, thereby favoring the disassembly of the compact conformation of c-Abl and favoring the activated over the autoinhibited state of the protein [19, 20]. In fact, the disassembled c-Abl structure has been observed crystallographically, where the myristoyl site is empty and the SH3, SH2 and kinase domains form a linear array [20]. This disassembled and elongated structure is thought to be a specific activated conformation of c-Abl [20]. Recently published hydrogen-exchange mass spectrometry data provide further support for this postulated mechanism and show that conformational changes play a major role in c-Abl kinase regulation in solution [22].

The interactions between the myristoyl group and the C-terminal helix are crucial in stabilizing the bent conformation of the helix, as evidenced by the observed straight form of the C-terminal helix when the myristoyl site is empty [21]. Hence, it is reasonable to hypothesize that the displacement of the myristoyl group from the myristoyl site may lead to c-Abl activation. This paper will present two chemical series of small molecule c-Abl activators discovered in-house. Representative molecules from each chemical series have been co-crystallized with the c-Abl kinase domain; these co-crystal structures confirm that both series bind to the myristoyl site. Detailed analyses of the interactions of these two chemical series of small molecule c-Abl activators with the myristoyl site will be described, from which mechanisms of c-Abl activation by these small molecules will be proposed. Optimization of these small molecules guided by structural knowledge and molecular modeling will be presented. Finally, we will conclude with insights gleaned from the successful optimization of small molecule c-Abl activators.

## Methods

### Molecular docking

**Fig. 2:**
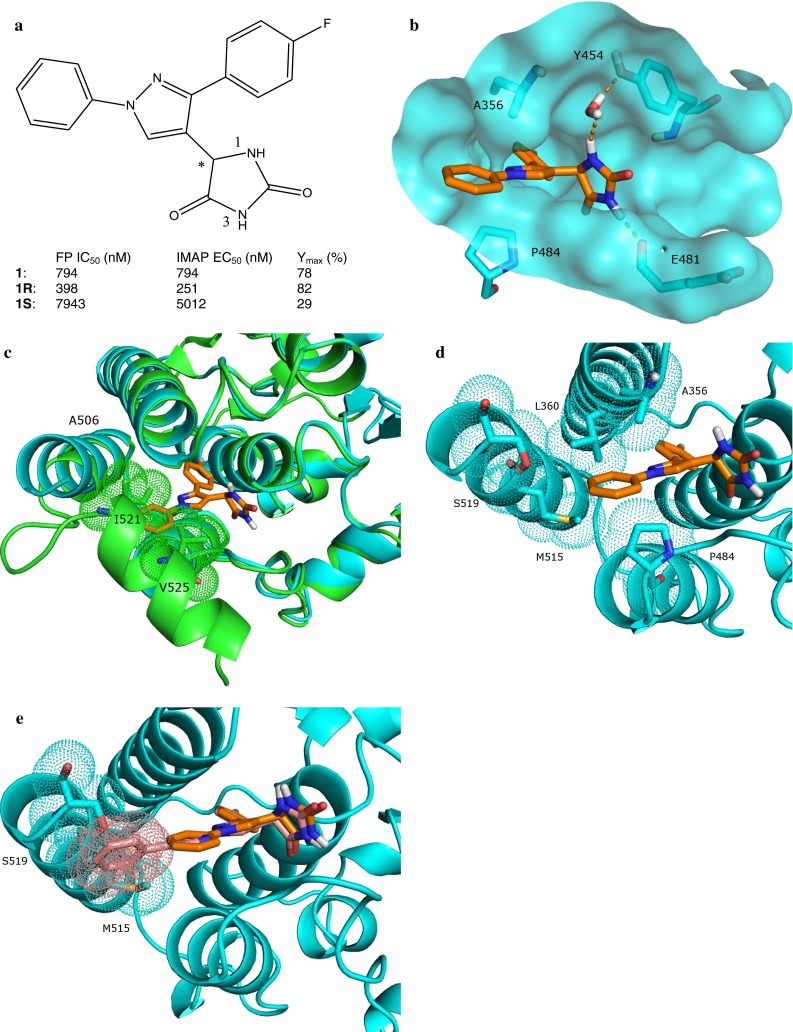
**a** Structures of pyrazole exemplars **1**, **1R** and **1S** shown with their FP IC_50_ and IMAP EC_50_ values. **b** Interactions of **1R** and the c-Abl myristoyl site (surface shown). For visual simplicity, only selected amino acids that interact with **1R** are shown, and they are shown in *cyan*. The carbon atoms of **1R** are in brown. Hydrogen bonds between **1R** and the myristoyl site are indicated with *dash lines*. **c** Overlay of 1R-bound and myristoyl-bound cAbl kinase domain. Ile521 and Val525 of the myristoyl-bond c-Abl are in *green* carbon. The surface of these two residues is also in *green*. **d** **1R** in the c-Abl myristoyl site. Key residues in the vicinity of **1R** LHS are shown along with their surface. **e** Overlay of the crystallographic conformation of **1R** and the docked pose of **3** in the c-Abl myristoyl site. The surface of Met515, Ser519, and the distal phenyl ring of **3** is shown in *cyan* and *pink*, respectively

**Fig. 3:**
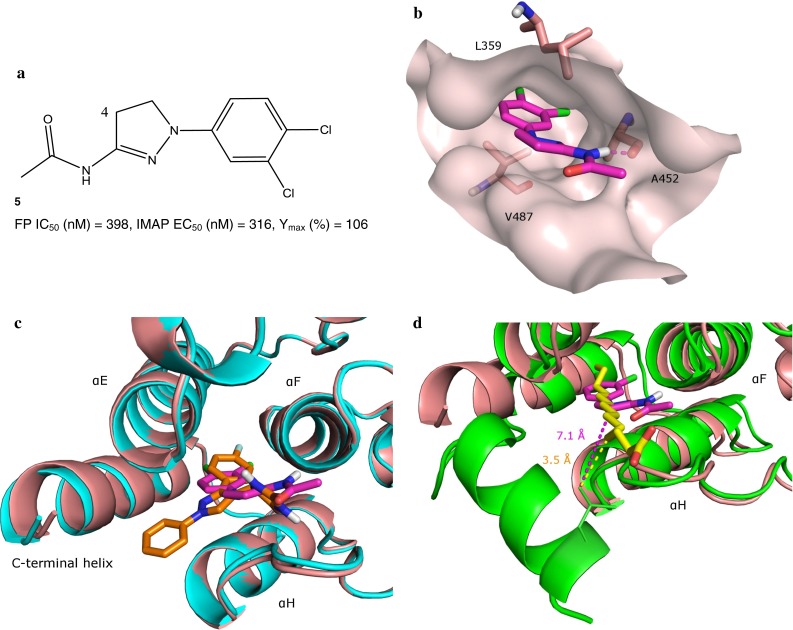
**a** Structure of dihydropyrazole exemplar **5** shown with its FP IC_50_ and IMAP EC_50_ values. Position 4 on the dihydropyrazole core is highlighted. **b** Interactions of **5** and the c-Abl myristoyl site (surface shown). For visual simplicity, only selected amino acids that interact with **5** are shown, and they are shown in *pink*. The carbon atoms of **5** are in *magenta*. Hydrogen bonds between **5** and the myristoyl site are indicated with *dash lines*. **c** Overlay of the c-Abl myristoyl site complexed to **1R** (shown in *cyan*) and **5**. **d** Overlay of **5**-bound and myristoyl-bound c-Abl kinase domains. The shortest distance between the alkyl chain of the myristoyl and the side chain of Leu529 is illustrated in a *yellow dash line*; while the shortest distance between a lipophilic atom of **5** and the side chain of Leu529 is shown in a *magenta dash line*

The co-crystal structures of **1R** (Fig. 2b) and **5** (Fig. 3b), the exemplar of each of the series, and the c-Abl catalytic kinase domain (248–531) were prepared using the protein preparation utility of maestro (version 2009.0.111), where bond orders were assigned, hydrogen atoms were added and waters that did not directly interact with the small molecules were removed. The exhaustive sampling option of the preparation utility was chosen to optimize the hydrogen bonding network of the complex structures. Then, a restrained minimization of coordinates of hydrogen atoms was conducted with the OPLS2005 force field and a converging RMSD of 0.3 Å. Designed analogs were drawn in Maestro and their geometries were optimized by the LigPrep program of Maestro with the OPLS2005 force field. They were docked to the c-Abl myristoyl site of either c-Abl—**1R** co-crystal structure or c-Abl—**5** co-crystal structure depending on which chemical series the analogs belonged to, using both Glide XP (version 5.5) and Gold (version 3.2). In preparation for Glide XP docking, receptor grids were generated by selecting either **1R** or **5** as the centroid of the binding site and applying default settings to remaining parameters. In running Glide XP, default parameters were utilized except that the threshold for removing a duplicate pose was set to 1 Å. For gold docking, search efficiency was set to 100 %. GoldScore was chosen as the scoring function. Ring corners, amide bonds and pyramidal N were allowed to flip. The internal H-bonds option was turned on. Default settings were used for remaining parameters. Resulting docked poses from Glide XP and Gold were combined and filtered by visual inspection.

To study the binding of **9** and **10** to the C-terminal helix in the bent conformation, the crystal structures of inactive c-Abl (PDB codes: 1OPK and 3K5V) were prepared with the procedure identical to that used to prepare the co-crystal structures of c-Abl bound to **1R** and **5**. **9** and **10** were also prepared in a way identical to what was described above. Glide SP was the docking engine. In preparation for Glide SP docking, receptor grids were generated by selecting either the myristoyl (in the case of 1OPK) or GNF2 (in the case of 3K5V) as the centroid of the binding site and applying default settings to remaining parameters. In running Glide SP, default parameters were utilized except that the threshold for removing a duplicate pose was set to 1 Å. Resulting docked poses of **9** and **10** in the myristoyl site in both the 1OPK and the 3K5V structures were combined and filtered by visual inspection.

### Identification of hydrophobic hotspots in the c-Abl myristoyl site

The MultiFragment Search (MFS) program implemented in MOE (version 2009) was used to probe the c-Abl myristoyl site for opportunities to improve interactions between the myristoyl site and the dihydropyrazoles, one of the two chemical series of small molecule c-Abl activators reported herein. The underlying methodology of the MFS program is multi-copy simultaneous search (MCSS) [23, 24]. In this method, multiple copies of fragments are randomly placed in a pre-defined binding site. The positions of fragment copies are minimized where each copy of a fragment only experiences the interactions with the binding site and the interactions between fragment copies are omitted. Local minima of fragment copies are located at the end of energy minimization, representing a functionality map of the binding site.

The crystal structure of the dihydropyrazole exemplar, **5** (Fig. 3b), complexed to the c-Abl kinase domain was prepared using the protocol described in the previous section. **5** was chosen as the centroid of the myristoyl site, and the residues within 6 Å from **5** were selected as binding site residues. Partial atomic charges of the binding site residues were assigned based on the Amber99 force field. Fragments used in the MFS search included methane, ethane, propane, butane, isobutene and cyclohexane. Their geometries were taken from the default fragment library of MOE and their partial atomic charges were assigned based on the MMFF94 force field. van der Waals radii of the fragments and the protein were scaled by 0.5 and 0.8, respectively, during the initial fragment placement step to allow close positioning of the fragments to the protein. Two-thousand copies of each fragment were randomly positioned in the c-Abl myristoyl site. Default settings were used for remaining parameters.

### Biological assays to measure c-Abl binding and activation of a small molecule

Details of the assays to measure c-Abl binding and activation of a small molecule have been described elsewhere [23, 31]. Briefly, c-Abl binding of a small molecule was measured in a fluorescence polarization (FP) competition binding assay, where the small molecule competed, upon binding to the c-Abl myristoyl site, against a carboxytetramethylrhodamine (TAMRA)-labeled peptide ligand that had the same sequence as c-Abl 1b N-terminus with a myristoyl group. Results were reported as FP IC_50_. c-Abl activation of a small molecule was measured in an immobilized metal affinity for phosphochemicals (IMAP) assay that determined the amount of c-Abl activation by quantifying the phosphorylation of a peptide, TAMRA–KKGEAIYAAPFA–NH_2_, that was used as the substrate of c-Abl. c-Abl activation results were reported as IMAP EC_50_. Maximal activation values fitted for small molecules (IMAP Y_max_) were reported as the percentage of c-Abl activity in the presence of the small molecule and 30 µM ATP relative to the control activity that was measured in the absence of the small molecule but in the presence of 90 µM ATP.

## Results

### Co-crystal structure of the pyrazole series and the c-Abl kinase domain

Pyrazole exemplar **1** (Fig. 2a) was discovered via high throughput screening (HTS) of the GSK compound collection. **1** is a racemic mixture of two enantiomers; the crystal structure of the more active **1R** enantiomer complexed to the c-Abl kinase domain was determined at 1.85 Å resolution (Fig. 2b). The interactions of **1R** and the myristoyl site have been described in detail previously [23], and a summary is provided below to facilitate the description of the modeling work conducted for the pyrazole series. The fluorophenyl ring of **1R** sits deeply in the inner hydrophobic pocket of the myristoyl site, where in addition to forming hydrophobic interactions with Ala363, Leu359, Leu448, Ile451, and Val487, the fluorophenyl ring also has a π–π stacking interaction with Phe512. The 1-NH of the hydantoin ring of **1R** is hydrogen-bonded to the side chain of Tyr454 via a mediating water molecule, while the 3-NH of the hydantoin has a direct hydrogen bond interaction with the backbone carbonyl of Glu481 (see Fig. 2a for the numbering scheme of the hydantoin ring). The pyrazole core of **1R** makes van der Waals contact with Ala356 and Pro484. The left-hand-side (LHS) phenyl ring extends towards the C-terminal helix and sits in a lipophilic groove with Ala356 and Leu360 lining the top and Pro484 and Val487 shaping the bottom (see Fig. 2b for visual simplicity, only selected amino acids that interact with **1R** are shown). c-Abl binding and activation values for **1**, **1R** and **1S** have been presented elsewhere [23] and are summarized in Fig. 2a. **1S** is significantly weaker in both binding and activation compared to **1R**. Docked poses of **1S** (data not shown) suggest that in the *S* configuration the hydantoin moiety is unable to form hydrogen bonds with the myristoyl site, which would thereby result in weaker binding and lower activation of c-Abl.

All helices except for the C-terminal helix in the **1R**-bound myristoyl site exhibit very little difference in conformation as compared to when the myristoyl is bound (Fig. 2c). With the residues on the C-terminal helix excluded, least-squares superposition of the c-Abl kinase domain complexed to **1R** and that to the myristoyl resulted in a RMSD of 0.6 Å for the residues within 6 Å from the bound conformation of **1R**. However, when only the residues on the C-terminal helix are considered, the RMSD is 5.6 Å. The **1R**-bound C-terminal helix starts to diverge from its myristoyl-bound counterpart at Ala506; this divergence continues for about two turns after which the helix becomes disordered. It is important to point out that the last crystallographically observable helical turn includes the five residues (i.e. Met515–Ser519) comprising the bend in the bent conformation of the C-terminal helix (Fig. 1b, c), and their electron density clearly demonstrates that the **1R**-bound C-terminal helix adopts an extended helical conformation. As discussed extensively in our previous publication [23], this conformational difference between the **1R**-bound and the myristoyl-bound C-terminal helix is likely due to the wedge-like shape of **1R** that makes it too bulky for the C-terminal helix to adopt the bent conformation. In particular, the overlay of the **1R**-bound c-Abl with the myristoyl-bound c-Abl shows that the LHS phenyl moiety of **1R** exhibits steric clash with Ile521 and Val525 from the third and second helical turn from last, respectively, of the C-terminal helix (Fig. 2c). The bent conformation of the C-terminal helix provides a necessary platform for the SH2 domain to dock onto and is crucial for keeping c-Abl in the autoinhibited state; we therefore hypothesize that inability to adopt the bent conformation would favor activation of the protein.

### Structure-guided optimization of the pyrazole series

The description above of the interaction between **1R** and the c-Abl myristoyl site indicates that the only obvious interaction of **1R** with the C-terminal helix is the π–π stacking between its fluorophenyl moiety and Phe512 of the helix. The LHS phenyl moiety of **1R** extends towards the C-terminal helix but its structure is not optimal to engage this helix. We hypothesized that extending the LHS would lead to increased interactions with the C-terminal helix. Examination of the myristoyl site surrounding the LHS pointed out that LHS extensions would target two residues on the C-terminal helix in particular: Met515 and Ser519. These two residues are from the last two turns of the straight form of the C-terminal helix with myristoyl-site-facing side chains (Fig. 2d). Met515 along with Ala356 and Leu360 of helice αE and Pro484 of helix αH form a hydrophobic surface surrounding the LHS phenyl of **1R**. While Ala356 and Pro484 are approximately half buried and Leu360 is completely buried by the binding of **1R**, Met515 remains partially solvent exposed. Its solvent-accessible surface area (SASA) is 11.1 Å^2^ when the myristoyl site is unoccupied and is reduced slightly to 9.7 Å^2^ upon **1R** binding. In comparison, Ser519 is more buried by the binding of **1R**. Its SASA reduces—from 98.5 Å^2^ in an unoccupied myristoyl site to 80.1 Å^2^ when **1R** is bound. However, a close inspection suggested that an extension of the LHS of the pyrazole scaffold might gain a hydrogen bond interaction with the side chain hydroxyl of Ser519; in addition, a lengthened LHS might also be able to increase hydrophobic contact with Met515. In designing LHS extensions, inspiration was drawn from the concept of small molecule α-helix mimetics (for a review of small molecule α-helix mimetics, see [24]). Our goal was not to disrupt α-helix-mediated protein–protein interactions; however, α-helix mimetics by principle would interact with helical secondary structures strongly, which was our design objective. In particular, di-phenyl was considered since it had been shown to allow a close spatial mimicry of the *i* and *i* + 3 or *i* + 4 positions of an α-helix. Phenyl bioisosteres were also considered and attempts were made to extend isosteres by a phenyl or another phenyl isostere. These α-helix mimetic scaffolds could then be elaborated to gain additional interactions with the C-terminal helix. As a proof of concept, analogs with unfunctionalized α-helix mimetic scaffolds as prolonged LHS were synthesized while taking synthetic tractability into consideration. Three exemplars are shown in Table 1.

**Table 1:**
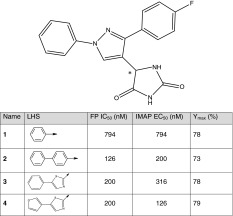
Structures of pyrazole analogs shown with their FP IC_50_, IMAP EC_50_ and IMAP Y_max_ values

The chiral center on the pyrazole template is highlighted by *. The pyrazole analogs listed in this table are racemic mixtures

Docked poses of these analogs suggested that lengthening of the LHS of the pyrazole scaffold would not alter the general interaction pattern of the pyrazole scaffold with the myristoyl site (Fig. 2e). The RMSD between the common moieties of the crystallographic conformation of **1R** and the docked poses of the designed analogs is <1 Å; these docked poses of the designed analogs are consistent with the hypothesis that an additional LHS aromatic ring would improve contact with Met515 and Ser519. Taking analog **3** as an example, the SASA of Met515 was predicted to reduce from 9.7 Å^2^ when **1R** was bound to 2.3 Å^2^ when **3** was bound, and the SASA of Ser519 was predicted to reduce from 80.1 Å^2^ when **1R** was bound to 54.3 Å^2^ when **3** was bound. Moreover, the docked pose of **3** suggested that, by adding a phenyl ring to its LHS, an OH-π hydrogen bond with Ser519 was achieved.

The designed analogs with lengthened LHS functionalities were synthesized as racemic mixtures and demonstrated nearly fourfold–sixfold increase over the racemic parent **1** in binding affinity to the myristoyl site (Table 1). This affinity increase translated nicely to c-Abl activation as measured by IMAP EC_50_. Little change in Y_max_ was observed, suggesting that the maximal activation of c-Abl by these analogs is similar to that induced by parent **1**. This observation is consistent with our design strategy where the focus was on increasing binding to the myristoyl site with the straight C-terminal helix and not on altering conformational states of the myristoyl site. The latter might produce more significant change in Y_max_. Chiral separation of these analogs was not performed; however, it is reasonable to expect that the active enantiomers of these analogs would exhibit approximately an additional twofold improvement in c-Abl binding over their racemic parents and further increase their c-Abl activation. Functionalization of prolonged LHS was not carried out due to re-allocation of chemistry resources. However, based on successful examples of elaborated α-helix mimetics reported in literature, it is conceivable that substituting LHS could augment c-Abl binding and activation even further.

### Co-crystal structures of the dihydropyrazole series and the c-Abl kinase domain

The dihydropyrazole series originated from hybridization of an in-house HTS-derived series with some hits from fragment screening [25]. The crystal structure of **5** (Fig. 3a), an exemplar of this chemical series, complexed to the c-Abl kinase domain was determined at 2.50 Å resolution (Fig. 3b for visual simplicity, only selected amino acids that interact with **5** are shown in this figure). Similar to **1R**, **5** binds to the c-Abl myristoyl site where the C-terminal helix again adopts a straight conformation with the last crystallographically observable residue also being Ser519. In addition, the myristoyl site does not exhibit significant conformational change upon binding to **5** as compared to **1R** (Fig. 3c). The overall RMSD of the c-Abl kinase domain bound to **1R** and to **5** is about 0.5 Å, and the RMSD of the residues within 6 Å from these two molecules is merely 0.6 Å. Similar to the fluorophenyl ring of **1R**, the dichlorophenyl moiety of **5** interacts with the inner hydrophobic pocket of the myristoyl site that is formed by the side chains of Ala363, Leu359, Leu448, Leu451, and Val487. Another similarity between the interactions that **1R** and **5** have with the c-Abl myristoyl site is that the dihydropyrazole group, resembling the pyrazole core of **1R**, has van der Waals contact with the side chains of Ala356, Leu359 and Pro484. On the other hand, hydrogen bonding patterns between **1R** and **5** are different. Instead of engaging Glu481 as 3-NH of the hydantoin of **1R** does, the amide nitrogen of **5** forms a hydrogen bond with the backbone carbonyl of Ala452. A water-mediated hydrogen bond with Tyr454 is observed in **1R** and is not repeated in **5**.

Perhaps the most significant difference in the way **1R** and **5** engage the c-Abl myristoyl site is that **5**, unlike **1R**, does not extend towards the C-terminal helix (Fig. 3c). Instead, it mainly interacts with αE, αF and αH, the three helices that, based on the overlay of the myristoyl-bound c-Abl with either **1R** or **5** bound c-Abl, undergo little conformational change. Moreover, the overlay of c-Abl bound to **5** and to the myristoyl (Fig. 3d) shows that **5**, unlike **1R**, would not clash with the bent conformation of the C-terminal helix. This presents a puzzle as to how the dihydropyrazoles stimulate c-Abl activation. The overlay of c-Abl bound to **5** and to the myristoyl reveals that the direction that **5** takes when approaching the opening of the myristoyl site diverges from that of the myristoyl. The acetamide moiety of **5** approaches the opening near the interface of helices αF and αH and away from the C-terminal helix, while the myristoyl approaches the opening from the center of the binding site. In addition, **5** is much shorter in length compared to the myristoyl. As a result, **5** has weaker interactions with the last three turns of the bent C-terminal helix than the myristoyl does. For example, Leu529 is the myristoyl-site-facing residue from the last turn of the bent C-terminal helix. The myristoyl has hydrophobic contact with the side chain of Leu529. The shortest distance between the alkyl chain of the myristoyl and the side chain of Leu529 is 3.5 Å (Fig. 3d). In comparison, the overlay of **5**-bound and myristoyl-bound c-Abl indicates that the shortest distance between **5** and the side chain of Leu529 is from the carbonyl oxygen of **5** to the side chain of Leu529 and is around 5.3 Å. The shortest distance between a lipophilic atom of **5** and the side chain of Leu529 is more than 7 Å. Consequently, **5** is expected to have a much weaker affinity to the last turn of the bent C-terminal helix than the myristoyl group does. Without a binding partner that provides stabilizing forces, the C-terminal helix would favor the straight conformation as evidenced by the observed straight form of the C-terminal helix when the myristoyl site is empty [21]. Since the bent conformation of the C-terminal helix is crucial for the compact autoinhibited conformation of c-Abl, **5** may be activating c-Abl simply by not providing interactions to stabilize the bent conformation of the C-terminal helix.

### Structure-guided optimization of the dihydropyrazole series

**Fig. 4:**
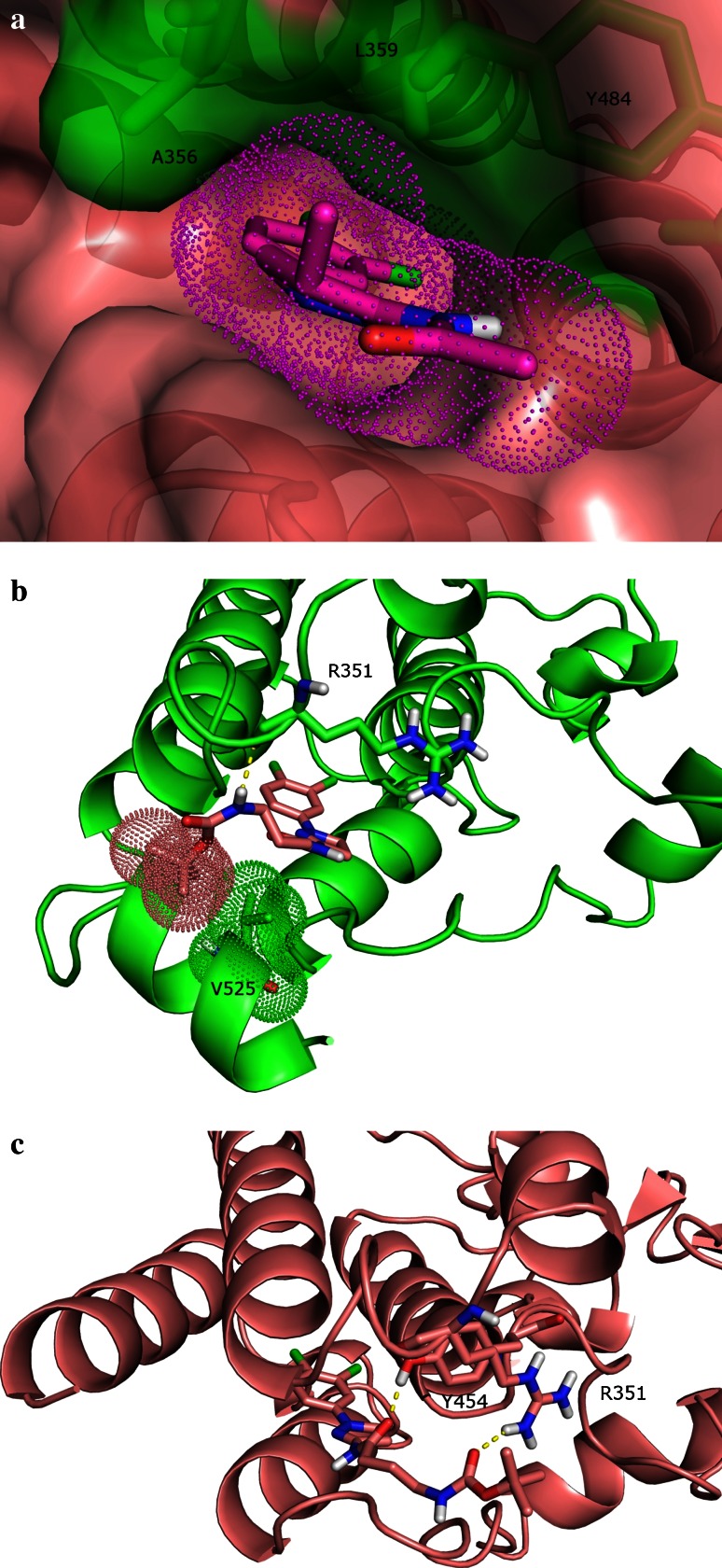
**a** A hydrophobic hotspot in the c-Abl myristoyl site identified by a multi-fragment search and the snug fit of dihydropyrazole analog **7** to this hotspot. The surface of the myristoyl site is shown in *pink*, with that of the hydrophobic hotspot in *green*. The three residues comprising the hotspot (i.e. Ala356, Leu359 and Tyr454) are rendered in stick format. The surface of the docked pose of **7** is shown in a *dotted* representation and is in *pink*. **b** The docked pose of **9** in the myristoyl site with the bent C-terminal helix. The surfaces of Val525 and *t*-butyl moiety of **9** are shown in *dotted* representations and are in *green* and *pink*, respectively. The hydrogen bond between **9** and Arg351 is represented by a *dash line*. **c** The docked pose of **9** in the myristoyl site with the straight C-terminal helix. The hydrogen bond between **9** and Y454 is represented by a *dash line*; so is the electrostatic interaction between 9 and Arg351

The postulated c-Abl activation mechanism laid out in the previous section for the dihydropyrazoles suggests that this series of molecules does not disrupt the bent C-terminal helix nor does it reinforce the bent conformation. The dihydropyrazoles may therefore act as merely myristoyl displacers. Therefore, the strategy to increase c-Abl activation for this chemical series focused on improving binding to the myristoyl site, making this series a better competitor of the myristoyl for the myristoyl site. To this end, a multi-fragment search [26, 27] was carried out to probe the myristoyl site for hotspots that the dihydropyrazoles could target to increase binding to the myristoyl site. This search revealed a hydrophobic hotspot largely composed of Ala356 and Leu359 of helix αE and Tyr454 of helix αF (Fig. 4a). This hotspot encompasses the dihydropyrazole ring. In addition, the multi-fragment search identified a low-energy position for a methane fragment that was about 1.8 Å away from the carbon atom at the 4 position of the dihydropyrazole ring (see Fig. 3a for the 4 position on the dihydropyrazole ring), which suggested that the portion of the hotspot that was not occupied already by the dihydropyrazole ring could be readily engaged by a small nonpolar moiety substituted at the 4 position of the ring. Furthermore, the multi-fragment search suggested that a substitution resulting in *S* stereochemistry at the carbon atom at the 4 position would engage the hotspot better than would an *R*-stereochemistry center. A methyl group was added at the 4 position of the dihydropyrazole core of **6** to test this hypothesis. This prediction is consistent with experimental data (Table 2); compared to **6** (*S*)-4-Me **7** exhibits threefold increase in c-Abl binding and over 12-fold improvement in c-Abl activation as measured by IMAP EC_50_, whereas (*R*)-4-Me **8** has reduced c-Abl binding and activation. The Y_max_ values of **7** and **8** are lower than that of parent **6**, but not significantly so. These comparable Y_max_ values suggest that the conformation of the myristoyl site predominately preferred by **7** and **8** is similar to that preferred by parent **6**, which is consistent with the rationale behind installing a methyl group onto the dihydropyrazole core. The relationship between preferred conformational states of the myristoyl site by small molecules and c-Abl activation is one of the main subjects of discussion in the remainder of this report.

**Table 2:**
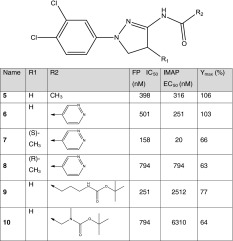
Structures of dihydropyrazole analogs shown with their FP IC_50_, IMAP EC_50_ and IMAP Y_max_ values

Another area for structural optimization was around the acetamide moiety of **5**. One might imagine that replacing the methyl with a more elaborate functional group would lead to additional interactions with the myristoyl site and benefit c-Abl binding and activation. An account of our efforts in this area is captured in a separate report [25]. We will focus the discussion herein on the impact that lengthening the dihydropyrazoles would have on c-Abl activation, a topic not covered in that separate report. As described previously, the length of the dihydropyrazoles played a key role in our hypothesis on the c-Abl activation mechanism of this series of compounds, and we were particularly interested to understand the impact of extension of the dihydropyraozles at the amide position. Two intermediates from a synthetic effort were specifically chosen to help address this question. The intermediates **9** and **10** are much longer than **5** and have terminal *t*-butyl groups (Table 2). Compared to **5**, **9** and **10** showed eightfold and 20-fold drop in c-Abl activation as measured by IMAP EC_50_, respectively, although Y_max_ remained largely unchanged. It was speculated that due to their length and terminal hydrophobic groups (i.e. the *t*-butyl group), a population of **9** and **10** might mimic the myristoyl group and also bind to the myristoyl site with the bent C-terminal helix. In other words, the myristoyl site with the straight C-terminal helix may not be the sole conformational state of the myristoyl site that **9** and **10** interact with. There may be favorable interactions that these two molecules could gain from engaging the myristoyl site with either the straight or the bent form of the C-terminal helix. Association of the bent-helix-containing myristoyl site with **9** and **10** would reduce the population of these two molecules available for binding to the myristoyl site with the straight C-terminal helix and, as a result, lead to reduced c-Abl activation for these two compounds.

To test this hypothesis in silico, **9** and **10** were docked to the myristoyl site with the bent C-terminal helix as well as the myristoyl site with the straight helix. (Since docking results of **9** and **10** are similar, we will focus the discussion on **9** for simplicity reasons.) The docked pose of **9** shows that in the myristoyl site with the bent C-terminal helix, the dichlorophenyl of **9** sits in the inner hydrophobic pocket of the myristoyl site, very much similar to the dichlorophenyl of **5** (Fig. 4b). The dihydropyrazole ring of **9** is predicted to flip and the nitrogen-containing side of the ring now face the C-terminal helix as compared to facing helices αF and αH in **5**. As a result of this flip, the hydrogen bond interaction between the amide and Ala452 no longer exists but a new hydrogen bond is formed between the distal amide nitrogen of **9** and the backbone carbonyl of Arg351. Interestingly, the *t*-butyl group of **9** is predicted to mimic the myristoyl group and form hydrophobic contact with Val525 from the second helical turn of the bent C-terminal helix. The closest carbon–carbon distance between the *t*-butyl moiety and the side chain of Val525 is about 4.3 Å.

Docking results of **9** in the myristoyl site with the straight C-terminal helix (Fig. 4c) show that the dichlorophenyl and the dihydropyrazole moieties would engage the myristoyl site in a similar manner as they do in the myristoyl site with the bent C-terminal helix (Fig. 4b). In the docked pose, the amide off the dihydropyrazole moiety forms a hydrogen bond with the side chain of Tyr454, and an additional electrostatic interaction is formed between the distal amide carbonyl of **9** and the guanidinium moiety of Arg351. The *t*-butyl moiety extends into the solvent while packing against the alkyl chain of Arg351.

A comparison of the docked poses of **9** in the two different conformational states of the myristoyl site shows that both binding events have favorable interactions such as hydrophobic interactions between the dichlorophenyl moiety and the inner hydrophobic pocket of the myristoyl site and hydrogen bonds to the residues near the opening of the myristoyl site. The hydrophobic contact between the *t*-butyl moiety and Val525 of the myristoyl site with the bent C-terminal helix is reminiscent of the contact seen between the alkyl chain of the myristoyl and this residue (Fig. 1b). On the other hand, favorable interactions both hydrophobic and electrostatic are predicted between the *t*-butyl moiety and the myristoyl site with a straight C helix as well. All in all, the docking results provide reasonable evidence that **9** can bind to the myristoyl site with either the bent or the straight C-terminal helix. Similar “promiscuous” binding is expected to account for the drop of c-Abl activation as measured by IMAP EC_50_ seen in **10** as well.

## Discussion and conclusion

While inhibition of protein kinase activity is often achieved by targeting the ATP binding site on the catalytic kinase domain by a small molecule, the discovery of kinase activators often requires an approach tailored towards a particular kinase’s activation mechanism [28]. The understanding of the activation mechanism for c-Abl kinase is greatly aided by the available crystal structures of autoinhibited and activated c-Abl. In the autoinhibited state, c-Abl adopts a compact conformation where the C-terminal helix of the kinase domain exhibits a bent conformation onto which the SH2 domain docks; this bent conformation of the C-terminal helix is stabilized by the N-terminal myristoyl group. In activated c-Abl, the myristoyl group is dislodged from the myristoyl site, eliminating interactions responsible for the formation of the bent conformation of the C-terminal helix. The straight C-terminal helix no longer provides the necessary interface for the SH2 domain to dock onto and the SH3, SH2 and kinase domains disassemble, which triggers the autoactivation cycle of c-Abl.

This understanding of the c-Abl activation mechanism provided important guidance to the optimization of two chemical series of small molecule c-Abl activators reported herein. Co-crystal structures of the c-Abl kinase domain with exemplars from these two chemical series show that both series bind to the myristoyl site where the C-terminal helix adopts the straight conformation. The overlay of the co-crystal structure of c-Abl bound to the first series, the pyrazoles, with the structure of the myristoyl-bound c-Abl shows that the pyrazoles would have steric clashes with the bent conformation of the C-terminal helix due to the wedge-like shape of this series of molecules and therefore would destabilize the bent C-terminal helix. The second series, the dihydropyrazoles, is linearly shaped and does not disrupt the bent C-terminal helix based on the overlay of the crystal structure of the dihydropyrazole-bound c-Abl with that of the myristoyl-bound c-Abl. However, molecules from this series do not reinforce the bent conformation either and act as merely myristoyl displacers. Optimization of c-Abl activation for the pyrazoles and the dihydropyrazoles focused on improving their binding to the myristoyl site with the straight C-terminal helix, essentially decreasing the chance of the myristoyl group from occupying the myristoyl site and inducing the straight-to-bent conformational change of the C-terminal helix. In particular, examination of the interactions of the pyrazole series and the myristoyl site indicated that lengthening of the LHS of the pyrazole template would increase contact with the straight C-terminal helix and increase c-Abl binding. Indeed, these modifications increased c-Abl activation of the pyrazoles by approximately sixfold. The discovery of a small hydrophobic hotspot in the myristoyl site aided the optimization of the dihydropyrazole series. A methyl group off the dihydropyrazole core was predicted to access this hotspot. When it was grafted onto the dihydropyrazole template, it delivered 12-fold boost in c-Abl activation and pushed the potency of this chemical series to double-digit nanomolar range.

Compounds **9** and **10** from the dihydropyrazole series impart important insight into the design of small molecules that regulate c-Abl activity via binding to the myristoyl site. The c-Abl activations of these two compounds are eightfold and 20-fold weaker than that of **5**, respectively. Structurally, these two compounds are much longer than **5**, the dihydropyrazole lead molecule, and have terminal hydrophobic groups. Docking suggests that **9** and **10** might exhibit promiscuous binding and possibly bind to both conformational states of the myristoyl site, one with the bent C-terminal helix and the other with the extended helix. This promiscuous binding would effectively reduce the population of **9** and **10** available to bind to the myristoyl site with the straight C-terminal helix and lead to reduced c-Abl activation for these two compounds. The Y_max_ values of **9** and **10** are lower than that of **5**, but not significantly so; we interpret this similarity in Y_max_ to mean that **9** and **10**, while binding to the myristoyl site with the straight or bent C-terminal helix, perhaps prefer the former conformation over the latter. As a consequence, the maximal activity of c-Abl while bound to **9** or **10** does not differ significantly from that of **5**-bound c-Abl. Perhaps, the more the small molecule favors the bent conformation of the C-terminal helix over the straight conformation, the weaker the small molecule’s c-Abl activation will be, and this weaker activation will be reflected in EC_50_ and increasingly in Y_max_ as well. Indeed, it was later discovered that a number of in-house small molecules showed single to double digit decrease in EC_50_ and significant drop in Y_max_ (more than twofold–fourfold) compared to **5**. Docking also suggested that these molecules exhibited non-selective binding to the myristoyl site, providing further support to the hypothesis proposed herein on **9** and **10**’s c-Abl activation. When structural modifications cause the small molecule to prefer the bent conformation predominately, these modifications would essentially turn the small molecule from a c-Abl activator to an allosteric c-Abl inhibitor. In fact, there are reports of small molecules that bind to the myristoyl site, mimic the myristoyl despite having distinct chemical structures from the myristoyl, and stabilize the bent conformation of the C-terminal helix [29, 30]; these reported small molecules have demonstrated inhibitory effects over c-Abl.

Hence, it is important not to focus solely on the straight C-terminal helix during the optimization of c-Abl activators. It may be less an issue if the activator demonstrates steric clashes with the bent C-terminal helix (for example, the pyrazole series), and the structural optimization does not remove moieties responsible for these clashes. But if the mode of action of the activator does not involve steric hindrance to the bent C-terminal helix and is simply myristoyl displacement (for example, the dihydropyrazoles), efforts should be spent to ensure designed molecules favor the straight over the bent form of the C-terminal helix during the optimization of these molecules’ c-Abl binding. Structure-based methods such as docking would provide valuable guidance. Designed molecules can be docked to the myristoyl site with the straight C-terminal helix as well as that with the bent helix. Careful examination of docked poses might help select the molecules that strongly favor the straight over the bent form of the helix. Once the molecules are synthesized, their preference of the conformation of the C-terminal helix can be elucidated by crystallography or biophysical techniques such as NMR, when weaker than expected c-Abl activation is observed. If the myristoyl site with the bent C-terminal helix is confirmed to be the predominant state that the small molecules bind to, structural optimization strategies for these molecules should be adjusted to decrease binding to the straight C helix.

To the best of our knowledge, this is the first publication where structural optimization of multiple series of small molecule c-Abl activators with distinct modes of action is presented. The insights reported herein may provide guidance to future design of small molecule c-Abl activators.

## Electronic supplementary material

Below is the link to the electronic supplementary material.

Supplementary material 1 (DOCX 12 kb)
