# Supplementary material for: Structure-guided optimization of small molecule c-Abl activators
Source: J Comput Aided Mol Des. 2014 Feb 27;28(2):75–87. doi: 10.1007/s10822-014-9731-5 (PMC3949015; doi:10.1007/s10822-014-9731-5)
Supplement: Supplementary file 1 — Supplementary material 1 (DOCX 12 kb) [file 10822_2014_9731_MOESM1_ESM.docx]

**Supplemental Table** Cellular activation (EC_50_) values of the compounds reported in this article.

| Name | Cellular activation EC_50_ (nM) |
| --- | --- |
| **1** | 2512 |
| **2** | 31623 |
| **3** | 31623 |
| **4** | 19953 |
| **5** | 794 |
| **6** | 501 |
| **7** | 251 |
| **8** | 15849 |
| **9** | 50119 |
| **10** | 31623 |

The structure-guided optimization strategies described in this article utilize mostly *in vitro* biological data including FP IC_50_, IMAP EC_50_, and IMAP Y_max_. The main motivation for using *in vitro* data was ease of interpretation. Cellular activation values could be influenced by many factors that are not related to specific c-Abl interactions such as membrane permeability and promiscuous binding to other proteins, etc. These factors would make it difficult to decipher activation mechanisms of small molecule c-Abl activators reported herein. Nonetheless, cellular activation data are provided in this table as a reference.
